# Supplementary material for: Feasibility, safety, and efficacy of intraoperative magnetic resonance imaging-guided hepatectomy for small hepatocellular carcinoma: A retrospective study
Source: PLoS One. 2024 Jun 28;19(6):e0306307. doi: 10.1371/journal.pone.0306307 (PMC11213349; doi:10.1371/journal.pone.0306307)
Supplement: Attachment — Submitted filename: renamed_eab78.docx [file pone.0306307.s001.docx]

Manuscript. Number: PONE-D-24-13064

Title: Feasibility, safety, and efficacy of intraoperative magnetic resonance imaging-guided hepatectomy for small hepatocellular carcinoma: A retrospective study

Journal: *PLOS ONE*

**Reviewer #1**

**Reviewer#1 point #1:**

Most patients with HCC <2cm we would not perform an open hepatectomy on today in the United States. Are the authors arguing the earlier resection of HCC <2cm will lead to better outcomes? If so, what are the survival and morbidity outcomes for these patients?

**Author response #1:**

Thank you for your suggestion. We performed iMRI-guided hepatectomy for lesions that were undetectable via preoperative Sonazoid CEUS, which were likely to be unresectable. It is not possible to directly compare the long-term postoperative outcomes of these small lesions with those that have grown sufficiently large to be detectable through preoperative modalities. Considering reports that the prognosis after radical resection for early HCC (including BCLC Stage 0) is favorable and scoring systems indicating that a tumor diameter of ≥2 cm is a prognostic risk factor, performing curative resection for early-stage HCC can be considered one of the treatment options. Therefore, if resection is possible, it can be used as one of the treatment options. (We have added this information to the Discussion p.20, lines 344–351 and References #29,30 in green text). Regarding multiple lesions, we believe that achieving curative resection is more desirable than compromising curability resulting from the inability to resect these small lesions. We believe long-term outcomes are also an important point; however, we have not been able to analyze them thus far. Therefore, this remains a subject for future investigation.

**Reviewer#1 point #2:**

What is the cost effectiveness of utilizing an iMRI vs. serial monitoring and f/u with imaging prior to resection once the HCC reaches a larger size? What are the survival outcomes of such as well? The authors discuss tumor free margins but no long-term outcomes.

**Author response #2:**

Thank you for your question. Although iMRI-guided hepatectomy is indeed costly, including the investment in equipment, it has the advantage of ensuring patient curability. However, we have not been able to analyze the long-term outcomes thus far; therefore, this remains a subject for future investigation, as mentioned in the limitations section.

**Reviewer#1 point #3:**

This study is obviously in comparison to pre-operative ultrasound but what about intra-operative ultrasound? We often confirm all liver lesions with intra-operative ultrasound as it is cheap and widely available at our institution.

**Author response #3:**

Thank you for your question. As you mentioned, intraoperative ultrasound is an extremely important tool in hepatectomy for liver tumors, and we routinely use it in all hepatectomies. However, there are small liver tumors that occasionally remain undetected during intraoperative ultrasound examinations. As we mentioned in Table 2 and Discussion (p. 22, lines 382–384), the identification rate of contrast-enhanced intraoperative ultrasonography (CEIOUS) of the target lesions was 36.0%, and 64.0% of them could not be undetectable and unresectable in a usual manner. iMRI may be potentially effective compared with CEIOUS for target lesions in this study; however, further investigation is needed.

**Reviewer#1 point #4:**

If the HCCs are <2cm, why were these performed open vs. minimally invasive?

**Author response #4:**

Thank you for your question. We believe that laparoscopic surgery is not necessarily essential because the long-term outcomes are comparable between open and laparoscopic surgery. However, we also believe that minimally invasive surgery is preferable, and whether it can be applied to laparoscopic surgery is worth exploring, including the development of new devices. (We have added this information to Discussion, p.22, Lines 390–392 in green text)

**Reviewer #2**

**Reviewer#2 point #1:**

Please use <http://www.ncbi.nlm.nih.gov/mesh>

**Author response #1:**

Thank you for your suggestion. We have updated the keyword to match the MeSH terminology.

**Reviewer#2 point #2:**

Lack of the abbreviations section.

**Author response #2:**

Thank you for your pointing this out. We have added a list of abbreviations to the end of the manuscript in red text.

**Reviewer#2 point #3:**

Lack of figures?

**Author response #3:**

Thank you for your question. It does not appear that there were any missing figures; however, we have uploaded them again.

**Reviewer#2 point #4:**

The reference section contains too many old references.

**Author response #4:**

Thank you for your pointing this out. We have updated the references by replacing the older ones with newer ones in red text. (Reference #6, #7, #17, #20, and #24)

**Reviewer #3**

**Reviewer#3 point #1:**

The work presented by you is commendable and gives insight into a new modality of treatment for small HCCs of the liver. The manuscript is well written in a logical flow. The results have been written in an organized way discussion is quite relevant. Conclusion is well written.

**Author response #1:**

Thank you for your positive feedback. We have made revisions based on the comments from other reviewers, and we would appreciate it if you could review the revised sections.
